# The Role of Immune Checkpoint Receptors in Regulating Immune Reactivity in Lupus

**DOI:** 10.3390/cells8101213

**Published:** 2019-10-08

**Authors:** Kun-Lin Lu, Ming-Ying Wu, Chi-Hui Wang, Chuang-Wei Wang, Shuen-Iu Hung, Wen-Hung Chung, Chun-Bing Chen

**Affiliations:** 1 Chang Gung Memorial Hospital, Linkou 333, Taiwan; johnlu710999@gmail.com (K.-L.L.); mini_robots@yahoo.com.tw (M.-Y.W.); , kiruamairo@gmail.com (C.-W.W.); hungshueniu@gmail.com (S.-I.H.); 2 College of Medicine, Chang Gung University, Kwei-Shan, Taoyuan 333, Taiwan; 3 Department of Dermatology, Drug Hypersensitivity Clinical and Research Center, Chang Gung Memorial Hospital, Taipei 105, Taiwan; 4 Cancer Vaccine and Immune Cell Therapy Core Laboratory, Chang Gung Immunology Consortium, Chang Gung Memorial Hospital, Linkou 333, Taiwan; 5 Whole-Genome Research Core Laboratory of Human Diseases, Chang Gung Memorial Hospital, Keelung 204, Taiwan; 6 Immune-Oncology Center of Excellence, Chang Gung Memorial Hospital, Linkou 333, Taiwan; 7 Department of Dermatology, Xiamen Chang Gung Hospital, Xiamen 361000, China; 8 Graduate Institute of Clinical Medical Sciences, College of Medicine, Chang Gung University, Kwei-Shan, Taoyuan 333, Taiwan

**Keywords:** autoimmunity, co-stimulatory signals, co-inhibitory signals, immune checkpoint, immune regulation, immune-related adverse events, systemic lupus erythematosus

## Abstract

Immune checkpoint receptors with co-stimulatory and co-inhibitory signals are important modulators for the immune system. However, unrestricted co-stimulation and/or inadequate co-inhibition may cause breakdown of self-tolerance, leading to autoimmunity. Systemic lupus erythematosus (SLE) is a complex multi-organ disease with skewed and dysregulated immune responses interacting with genetics and the environment. The close connections between co-signaling pathways and SLE have gradually been established in past research. Also, the recent success of immune checkpoint blockade in cancer therapy illustrates the importance of the co-inhibitory receptors in cancer immunotherapy. Moreover, immune checkpoint blockade could result in substantial immune-related adverse events that mimic autoimmune diseases, including lupus. Together, immune checkpoint regulators represent viable immunotherapeutic targets for the treatment of both autoimmunity and cancer. Therefore, it appears reasonable to treat SLE by restoring the out-of-order co-signaling axis or by manipulating collateral pathways to control the pathogenic immune responses. Here, we review the current state of knowledge regarding the relationships between SLE and the co-signaling pathways of T cells, B cells, dendritic cells, and neutrophils, and highlight their potential clinical implications. Current clinical trials targeting the specific co-signaling axes involved in SLE help to advance such knowledge, but further in-depth exploration is still warranted.

## 1. Introduction 

A balanced immune system is vital for good health, as diminished immunity cannot protect us from various infections and malignancies, whereas skewed or unrestricted inflammation leads to autoimmunity and devastating collateral damage. Ultimately, the necessary balance relies on organized and timely communications between stimulatory and inhibitory pathways in the immune system. In general, an adaptive immune response is triggered by the antigen presented by the antigen-presenting cells (APCs), followed by subsequent interactions between different immune cells. These processes involve a variety of co-signaling and cytokine receptors, and together determine the end results of the immune response. While co-stimulatory receptors play essential roles in relaying the response, co-inhibitory receptors are generally induced following stimulations and subsequently transduce signals that moderate the co-stimulatory signals. Additionally, the existence of compensatory mechanisms is also suggested based on the findings of previous interventional studies [1], though the crosstalk among different axes largely remains to be explored. Given the complexity of the immune system, fruitful results may only be obtained from the manipulation of these co-signaling axes after their specific roles have been clearly elucidated.

Systemic lupus erythematosus (SLE) is one of the most devastating autoimmune diseases and is known for its complicated and skewed immune responses, including autoantibody formation, immune complex deposition, and cytokine activation [2]. Although the etiologies and clinical presentations of SLE are also complex, it is normally believed to be caused by the loss of self-tolerance with excessive activation of autoreactive T cells which subsequently promotes autoantibody production by auto-B cells along with excessive expressions of pro-inflammatory cytokines that further enhance the immune response [3]. Dendritic cells (DCs) are also crucial in the modulation of peripheral tolerance to self-antigens [4,5]. Neutrophils have been linked to the pathophysiology of SLE, and the release of neutrophil extracellular traps (NETs) during a distinct process of cell death, known as NETosis, plays an important role in the tissue damage experienced by patients with SLE [6]. These immune cells and an augmented expression of co-stimulatory molecules are thought to be critical for the disease pathogenesis of SLE. 

Notably, the recent success of immune checkpoint blockade in cancer therapy illustrates the importance of two inhibitory pathways, cytotoxic T-lymphocyte-associated antigen 4 (CTLA-4), and programmed cell death protein 1 (PD1) and its ligands (PD-L1, PD-L2), in the regulation of anti-tumor immune responses. Relatedly, the blocking of these inhibitory immune checkpoint receptors is also associated with further immune-related adverse events (irAEs) that can resemble autoimmune rheumatic diseases, including SLE [7]. Accumulating evidence has further suggested that dampening immune responses by either blocking the co-activating signals or enhancing the co-inhibitory signals in different cell types is a promising approach to treating autoimmune diseases. Herein, we review the most up-to-date literature regarding the co-signaling pathways of T cells, B cells, and dendritic cells, as well as neutrophils, involved in the pathogenesis of SLE. We also focus on the outcomes of the development of clinical trials targeting these pathways and discuss the primary challenges to further advances that remain.

## 2. Co-Signaling Axes in T Cells Relating to SLE 

SLE is generally believed to involve the breach of tolerance of CD4+ T cells, leading to subsequent autoreactive immune responses as well as an abnormal tendency toward inflammation. Although the precision of the immune system mainly relies on the specific recognition of antigens presented by major histocompatibility complex (MHC) molecules ligating the T cell receptor (TCR), co-stimulatory and co-inhibitory receptors on T cells are believed to collectively determine the fate of those T cells [8]. 

### 2.1. Involvement of Co-Stimulatory Receptors on T Cells in SLE

#### 2.1.1. CD28 

**Figure 1:**
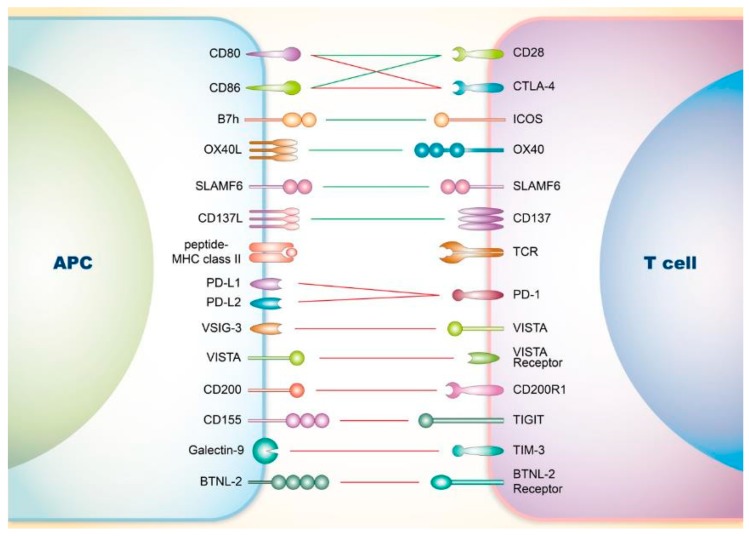
Co-signaling axes of T cells driving systemic lupus erythematosus (SLE). T cell is initially activated by the bridging between the T cell receptor (TCR) and major histocompatibility complex (MHC) on the surface of antigen-presenting cells (APCs). Besides this first signal, T cell activity could further be regulated by multiple co-stimulatory as well as co-inhibitory axes that participate in the cell-cell interaction. Co-stimulatory axes (green line) facilitate successful activation of T cells, whereas co-inhibitory axes (red line) limit the activation. CTLA-4: cytotoxic T-lymphocyte-associated antigen 4; ICOS: Inducible co-stimulator; SLAMF6: signaling lymphocyte activation molecule family 6; TCR: T cell receptor; PD-1: programmed cell death protein 1; VISTA: V-domain Ig suppressor of T cell activation; TIGIT: T-cell immunoreceptor with Ig and ITIM domains; TIM-3: T-cell immunoglobulin and mucin-domain containing-3.

Since the discovery of CD28, the receptor for CD80 (B7.1) and CD86 (B7.2) proteins, as a prototype co-stimulatory receptor on T cells, the well-known two-signal model of T cell activation has been recognized, highlighting the fact that both TCRs and co-stimulatory signaling are essential for full T cell activation [9,10,11]. Since then, various co-stimulatory pathways have been discovered (Figure 1), and some of them have been successfully utilized in treatments against autoimmune diseases as well as malignancies. 

CTLA-4 is a cell surface molecule that is closely related to CD28, and it has been reported to also be a powerful negative regulator of T cell activation [12]. By targeting the CD80 and CD86 molecules through CTLA-4 chimera proteins, both abatacept and belatacept became the earliest approved treatments against various autoimmune diseases, including rheumatic arthritis and juvenile idiopathic arthritis [13,14]. With respect to SLE, there are various forms of evidence supporting the rationale of treating SLE patients via the manipulation of this pathway. The polymorphism at the CTLA-4 promoter has been identified as being related to susceptibility to SLE [15]. CTLA-4 also modulates humoral responses by affecting follicular helper T (Tfh), follicular regulatory T (Tfr), and regulatory T (Treg) cells [16]. Moreover, CTLA-4–Fc has been proven to be highly effective in mouse models of lupus [17,18]. The clinical efficacy of CTLA-4 chimera proteins has likewise been tested in SLE patients. However, the results of the clinical trials completed thus far were disappointing as two phase II trials and one phase III trial failed to meet predetermined endpoints [19,20,21]. BMS-931699, an anti-CD28 protein, has also been investigated in a phase II clinical trial to determine its effects against SLE, but the results of that trial also failed to meet the hoped for endpoints [22]. Despite the negative results so far, however, there are an additional two phase II trials of abatacept currently under recruitment to further verify its efficacy [23,24].

#### 2.1.2. Inducible Co-Stimulator (ICOS) 

Inducible co-stimulator (ICOS), which is recognized as the third member of the CD28 family, serves as a co-stimulatory receptor of T cells that is essential for their activation and can further promote downstream humoral immunity [25]. It is expressed after the activation of naive T cells, which is dependent on prior CD28 signaling [26,27]. The ligand for ICOS, B7h (also known as B7RP-1), has been demonstrated to be constitutively expressed on B cells and macrophages, whereas inflammatory stimuli can induce its expression in non-lymphoid tissues as well as certain fibroblasts [28,29]. In previous murine studies, the generation, function, and maintenance of Tfh and extra-follicular Th cells that facilitate germinal center (GC) formation, B cell maturation, and IgG production were found to be dependent on ICOS [30,31]. A recent study on lupus-prone mice further elucidated that the systemic inflammation in lupus critically depends on ICOS stimulation by DCs and CD11c+ macrophages via the induction of essential PI3K-mediated pro-survival signals in organ-infiltrating T cells [32]. In addition, the suppression effect against glomerulonephritis in lupus-prone mice by nasal anti-CD3 treatment was found to be associated with a significant reduction in the percentage of IL-17 expressing CD4+ICOS+CXCR5+ T cells [33]. Moreover, previous studies have noted that in contrast with the aforementioned B7.1/B7.2-CD28/CTLA-4 and programmed death-ligand (PDL) 1/PDL2-programmed cell death protein 1 (PD-1) axis, B7h and ICOS are the only counterparts for each other [34]. Therefore, targeting this pathway with AMG557, a human Ab that targets the ICOS ligand, may be effective against SLE. To date, a phase I clinical trial of AMG557 has shown that it has an acceptable safety profile [35], but a clear answer as to whether its efficacy can be translated into clinical scenarios will require further explorations. 

In addition to the B7 family discussed above, co-inhibitory signals of B7-H3, BTNL2, and B7S1 have also been shown to negatively regulate T cell functions [36,37,38]. Intriguingly, previous studies have revealed that CD28 co-stimulation greatly increased the proliferation of B7-H3-treated T cells, did so less well in BTNL2–Ig-treated cells, and had no effect on cells stimulated with B7S1–immunoglobulin (Ig) [25]. Moreover, IL-2 and CD28 co-stimulation synergistically enhanced T-cell proliferation in the presence of B7-H3–Ig or BTNL2–Ig but not B7S1–Ig [25]. These results suggested that different mechanisms are utilized by these three molecules in T-cell regulation. Therefore, despite the finding that BTNL2 polymorphism exhibits strong linkage disequilibrium with autoimmune diseases including SLE [39], it is still important to carefully examine whether targeting this pathway could overcome the effects of other co-stimulation signals.

#### 2.1.3. OX40 

Besides the co-stimulatory pathways tested out in clinical trials, there are many other axes that have shown immunomodulatory potential (Table 1). In preclinical studies of SLE, one of the most studied co-stimulation axes has been that consisting of the OX40 (also known as TNFRSF4 or CD134) expressed by activated T cells along with its ligand OX40L. To be specific, OX40 can promote T helper (Th) cell survival [40], cytokine production [41], and T cell accumulation within B cell follicles [42], as well as memory cell formation [43], while it also participates in the regulation of the balance of Treg, effector T, and Th cell functions [44,45]. Previous murine studies have revealed that the OX40-OX40L axis is crucial in the development of autoimmune diseases, and that disrupting this axis could be utilized to prevent and treat these diseases [46]. It was further discovered that OX40L can stimulate Tfh responses by activating OX40L+ APCs, contributing to SLE pathogenesis [47]. Furthermore, it was reported that polymorphism in OX40 correlates with increased susceptibility to SLE [48]. Moreover, in addition to OX40 and OX40L having been found to be abundant in the glomerular walls of proliferative lupus nephritis patients, the expression of OX40 on peripheral blood T cells from patients with lupus nephritis has been found to be correlated with disease severity [49,50,51]. These findings have indicated the therapeutic potential of anti-OX40 therapy. Relatedly, the in vitro treatment of splenocytes from lupus-prone BXSB mice with OX40L mAb, in combination with an anti-CTLA-4 strategy, suppresses autoantibody production and pro-inflammatory cytokines [52]. Similarly, although a high percentage of IL-10 secreting cells has been noted in patients with lupus nephritis, in vitro treatment of their peripheral blood mononuclear cells with anti-OX40 therapy reduces IL-10 expression [53]. Additionally, in a recent study of a type I interferon-accelerated NZB/NZW.F1 mice model, anti-OX40 treatment significantly delayed severe proteinuria onset and improved survival [54], suggesting the potential therapeutic benefit of targeting this axis in SLE.

#### 2.1.4. Signaling Lymphocyte Activation Molecule Family (SLAMF) 

It is important to point out that the role of the co-stimulatory receptors of the signaling lymphocyte activation molecule family (SLAMF) in SLE has also been of considerable interest recently because of their vital role in Tfh cell function [55]. Multiple genome-wide association studies of families with multiple members affected with SLE have found a susceptibility locus which includes the SLAMF genes [56]. Furthermore, SLAMF3 and SLAMF6 receptors on T cell surfaces have been reported to be positively associated with disease activity in SLE patients, possibly via increasing IL-17 production [57]. Moreover, signaling via SLAMF6 also enhances Th1 cytokine production, likely through clustering with TCR and increasing T cell adhesiveness [58], but this effect has been shown to be defective in SLE patients [59]. Other SLAMF receptors have also been suggested to play roles in both murine models of SLE and SLE patients [60,61]. For instance, SLE patients were found to have enhanced expressions of SLAMF1 on both T cells and B cells, whereas SLAMF2 level was increased on their CD4+ and CD8+ T cells [62]. In addition, lupus nephritis (LN) patients which were not responded to B cell depletion therapy had reported to have a higher proportion of SLAMF6 expression on CD4− CD8− T cells. Moreover, CD8+ T cells expressing SLAMF3, SLAMF5, and SLAMF7 were all significantly decreased in LN patients who were in remission [63]. Further clarification of their roles in SLE is essential before it is possible to utilize them to treat SLE in clinical scenarios.

#### 2.1.5. CD137

As another co-stimulating pathway, CD137 (also known as TNFRSF9 or 4-1BB) belongs to the TNF/TNF receptor family in T cells, but it has also been found on a variety of immune cells, including B cells, natural killer (NK) cells, DCs, neutrophils, and monocytes [64]. CD137 signaling not only biasedly enhances the proliferation and survival of CD8+ T cells but also promotes IL-2 production by CD4+ T cells while preventing activation-induced cell death [65,66]. However, possibly due to the fact that anti-CD137 mAbs have multiple targets, their perplexing in vivo roles are still under investigation. For instance, the deletion of CD137 ligand of lupus-prone mice worsens their renal and cutaneous manifestations of lupus but lessens SLE-related neurological damage [67]. As another example, it was found that CD137–/– of lupus-prone mice resulted in increased levels of serum anti-dsDNA autoantibodies, Ig deposition, the accumulation of pathogenic T cells, and the exacerbation of both skin lesions and lacrimal gland inflammation [68,69]. On the other hand, agonistic anti-CD137 mAb treatment of NZB/NZW.F1 mice suppresses GC formation and anti-dsDNA IgG production without inducing immunosuppression, reversing SLE and prolonging the mouse’s lifespan [70]. The ability to dampen overall humoral immunity and therefore extend survival by targeting this axis was also confirmed in another lupus-prone mouse model treated with αCD137 Ab [71], and the suppressed CD4+ T-dependent humoral immune responses may be responsible for these findings [72]. 

### 2.2. Involvement of Co-Inhibitory Receptors on T Cells in SLE

CTLA-4 and PD-1/PD-L1 are the two most well studied co-inhibitory pathways in terms of targeting such pathways to fight malignancies [73]. Immune checkpoint inhibitors with anti-CTLA-4 antibodies (Abs) and anti-PD-1/PD-L1 Abs have been approved by the U.S. Food and Drug Administration for the treatment of metastatic non-small cell lung cancer, melanomas, head and neck squamous cancers, urothelial carcinomas, gastric adenocarcinomas, etc. [74,75,76]. However, while these therapies have achieved clinical success in patients with various malignancies, blockades of CTLA-4 and PD-1/PD-L1 are associated with side effects known as irAEs that can resemble autoimmune disorders, including SLE, rheumatic arthritis (RA), thyroiditis, Stevens–Johnson syndrome/toxic epidermal necrolysis, colitis, pneumonitis, myocarditis, type 1 diabetes, etc. [7,77,78]. In contrast, the augmentation of these co-inhibitory axes holds the potential to stop the progression of autoimmune diseases. 

#### 2.2.1. CTLA-4

As one of the most promising approaches against malignancy, anti-CTLA-4 treatment (such as ipilimumab, tremelimumab) has been extensively utilized in clinical scenarios, but its irAEs had raised substantial concerns. It is known that CTLA-4 deficiency promotes preferential expansion of Treg cells, leading to subsequent non-infectious inflammation likely through the production of organ-specific autoantibodies [79,80,81]. A recent meta-analysis demonstrated that the overall incidence of irAEs associated to anti-CTLA-4 treatment was 72% for all-grade and 24% for high-grade [82]. Among the irAEs related to checkpoint inhibitors, the incidence of rheumatic manifestations approximately accounts for 3.5% of all patients treated, with the majority of them being inflammatory arthritis [83]. Interestingly, lupus has rarely been reported as an irAE of checkpoint inhibitors [84], reflecting its complex nature that may require a multi-faceted approach based on comprehensive understandings of the pathogenic axes involved.

In the previous section, we introduced how CTLA-4 chimera proteins could compete with CD28 for CD80 and CD86, therefore inhibiting T cell responses. To our knowledge, there had not been a therapeutic strategy for SLE by enhancing the CTLA-4 responses, but similar approaches were investigated extensively in other axes. 

#### 2.2.2. PD-1 

PD-1 belongs to the surface protein that can bind to its ligand and inhibit the proliferation and function of T cells. PD-1 receptor is expressed after T cell activation. PD-1 interacts with two ligands, PD-L1 and PD-L2. PD-L1 is also expressed on the surface of APCs, as well as epithelial and endothelial tissues [85], whereas PD-L2 is expressed mainly by APCs. The expression of PD-L1 inhibits the proliferation of activated T cells. Anti-PD-1 (such as nivolumab, pembrolizumab) and anti-PD-L1 antibodies (atezolizumab, durvalumab, avelumab) prevent PD-1/PD-L1 and PD-1/PD-L2 binding and result in the restoration of the activity of antitumor T cells [86]. Similar to anti-CTLA treatment, these anti-PD1/anti-PD-L1 agents also cause irAEs with variable autoimmune disorders. 

PD-1 has been extensively explored in preclinical studies of autoimmune diseases. PD-1 mRNA is known to be broadly expressed at low levels in T, B, and myeloid cells, and could be further upregulated upon activation [87]. PD-1 deficiency in mice leads to spontaneous, lupus-like autoimmune diseases, and the introduction of the lymphoproliferation (lpr) mutation promotes lupus onset [88,89]. It is also known that both deficiency and blockade of PD-1 accelerate autoimmune diabetes in non-obese diabetic mice, while blocking PD-1 was found to induce experimental autoimmune encephalomyelitis (EAE) in mice [90,91,92]. However, controversial results were found in targeting PD-1 by the fusion proteins of its ligands, PD-L1–Ig and PD-L2–Ig. Both fusion proteins have been shown to either stimulate or inhibit CD4+ T cell responses [89,93]. Moreover, whereas blockade with anti-PD-L1 Abs accelerates the onset of LN, PD-1 blockade, in contrast, limits LN and facilitates immunosuppression via both CD4+ and CD8+ Treg cells in NZB/NZW.F1 mice, one of the most widely utilized mouse models of SLE [94,95,96]. As a step forward to reconcile the inconsistencies in previous findings, a recent study revealed that the PD-1 pathway modulates Tfh-mediated humoral immunity by downregulating Tfr cells [97], which indicated that blocking PD-1 may preferentially influence the PD-1 function in Tfr cells and therefore mitigate lupus manifestations. Although polymorphisms at *PDCD1* have also been reported to be associated with susceptibility to SLE [98], the clinical efficacy of manipulating this pathway still requires further investigation based on the preclinical studies to date.

#### 2.2.3. V-Domain Ig Suppressor of T Cell Activation (VISTA)

In addition to the well-known pathways currently under investigation, the recent discoveries of several new axes have also brought new vigor and vitality to this field (Table 2). As a novel co-inhibitory axis, V-domain Ig suppressor of T cell activation (VISTA) is known to be expressed on T cells and some subsets of APCs. In vitro exposure to VISTA–Ig inhibits T cell proliferation and cytokine production, while blocking VISTA on mouse APCs enhances T cell responses [99]. Previous studies have further shown that VISTA-knockout mice are more susceptible to EAE [100], whereas both VISTA deficiency and blockade in SLE mouse models promote the activation of splenic CD4+ T cells and myeloid cell populations, resulting in increased pro-inflammatory cytokines, as well as more severe proteinuria and LN [101,102]. In terms of its therapeutic potential, a study based on the NZB/NZW.F1 mouse model of lupus has shown that the prophylactic use of VISTA–Ig prevents proteinuria and weight loss, while its therapeutic use also reverses proteinuria [103].

#### 2.2.4. CD200

Another co-signaling pathway affecting T cells, consisting of CD200R1 and its ligand CD200, is expressed on multiple immune cell types, including macrophages, neutrophils, monocytes, and subsets of T cells and B cells [7]. Their expression can be induced by chronic infection, regulating the inflammatory threshold, Th2 polarization, and immune homeostasis [104]. Previous studies on autoimmune diseases have further shown that the treatment of EAE and collagen-induced arthritis with CD200–Fc fusion protein reduces disease severity [105,106]. Meanwhile, a recent in vivo study of SLE based on NZB/NZW.F1 mice revealed that they have significantly lower percentages of CD200-CD200R1-positive cells in their splenocytes with significantly higher plasma anti-dsDNA levels that could be decreased after anti-CD200 treatment [107]. In another recent study of SLE patients, decreased expression of CD200R1 by CD4+ T cells and DCs was noted along with higher numbers of CD200+ cells and greater levels of soluble CD200 [108]. Moreover, the same study also found that in vitro engagement of CD4+ T cells with CD200 attenuated the differentiation of T-helper type 17 (Th17) cells and reversed the defective induction of a subset of Treg through transforming growth factor-beta, while anti-CD200R1 Ab facilitated CD4+ T-cell proliferation. Although the anti-inflammatory potential of CD200R1 agonist was demonstrated to limit the LPS-induced inflammation of human renal proximal tubular epithelial cells [109], the clinical efficacy of treating SLE or LN via this approach requires further elucidation. Interestingly, CD200R1 also plays a role in osteoclastogenesis without affecting osteoblast formation [110,111,112]. Therefore, besides its immunomodulatory function, targeting this axis may also have the potential to prevent bone destruction from various forms of autoimmune arthritis.

#### 2.2.5. T-Cell Immunoreceptor with Ig and ITIM Domains (TIGIT)

Recently, the co-inhibitory axis of T-cell immunoreceptor with Ig and ITIM domains (TIGIT) present on activated CD4+ T cells and NK cells has drawn great attention. Poliovirus receptor (PVR, also called CD155), a surface receptor highly expressed on DCs, fibroblasts, and some tumor cells, has high-affinity ligation to TIGIT [113,114,115]. It was found, moreover, that TIGIT+ CD4+ T cells exhibit a more activated phenotype than TIGIT− CD4+ T cells [116]. The frequency of TIGIT-expressing CD3+CD4+ T cells is significantly elevated in SLE patients, especially in severe cases [117]. Of note, these activated T cells with TIGIT+ do respond to co-inhibitory signals from TIGIT. Relatedly, recent in vitro studies found that activating the TIGIT pathway not only reduces the proliferation of T cells from mice, but also substantially down-regulates the activities of CD4+ T cells from SLE patients [116,118]. This could be explained by the fact that interactions between TIGIT and PVR on DCs lead to increased IL-10 secretion by DCs and, consequently, reduced proliferation of T cells. In line with these findings, in vivo studies revealed that targeting this axis could delay the development, or even improve the survival, of lupus mice [116,119]. Intriguingly, a significantly lower frequency of TIGIT+ NK cells was noted in SLE patients, and this phenomenon could be reversed after regular treatment [120]. However, whether this finding implies the potential synergistic effect of targeted therapy with regular treatment remains to be explored.

#### 2.2.6. T-Cell Immunoglobulin and Mucin-Domain Containing-3 (TIM-3) 

As a negative regulatory checkpoint shared by a variety of immune cells, T-cell immunoglobulin and mucin-domain containing-3 (TIM-3) and its ligand galectin-9 are best known for their high expressions in SLE patients [121,122,123] and have also been reported to be correlated with the activity of the disease [124,125]. With respect to the possible mechanisms by which TIM-3 and galectin-9 drive SLE, it was proposed that the elevated expression of soluble TIM-3 may impair the clearance of apoptotic cells [126], whereas the increase in galectin-9 may promote SLE by inhibiting the functions of regulatory T cells [127]. However, there had also been experiments showing that the intervention of lupus-prone mice with intraperitoneal galectin-9 could ameliorate their proteinuria and arthritis by decreasing the anti-dsDNA antibody levels, likely through the pro-apoptotic effect of galectin-9 on plasma cells [128]. Therefore, although selectively targeting this axis holds the potential to treat SLE, further investigations are required to elucidate their effects on different immune cells. 

#### 2.2.7. Others

So far, clinical trials investigating treatments of SLE focusing on the co-signaling pathways of T cells through direct cell-cell contact have generally failed to find success (Table 3). Many co-signaling axes of T cells associated with SLE that are still under investigation, including the herpes virus entry mediator-B- and T-lymphocyte attenuator (BTLA) signaling [129], CD94 ⁄NKG2A-HLA class I histocompatibility antigen, alpha chain E [130,131], and other axes, are not discussed at length in this review, but targeting their downstream signaling, as well as cytokine-mediated pathways, has shown encouraging results [132]. For instance, calcineurin is a phosphatase involved in facilitating TCR downstream signaling through multiple pathways [133,134,135,136], and its inhibition with cyclosporin A or tacrolimus is widely utilized for various immunosuppressive purposes [137,138]. A phase II randomized control trial of voclosporin, a chemical analogue of cyclosporin A that causes greater calcineurin inhibition, recruited 265 patients with active lupus nephritis, and demonstrated that both complete and partial renal response rates were significantly higher in the 2 voclosporin arms (23.7 or 39.5 mg bid) compared to the placebo group, in the background of glucocorticoid and mycophenolate mofetil (2 g/day) treatment [139]. These promising results have since led to a larger phase III global study of voclosporin (NCT03021499). Along with other positive findings for the treatment of SLE with tacrolimus plus mycophenolate mofetil combinations [140,141], these results again underline the potential of combinatorial therapies in treating SLE. 

## 3. Co-Signaling Axes in B Cells Relating to SLE 

**Figure 2:**
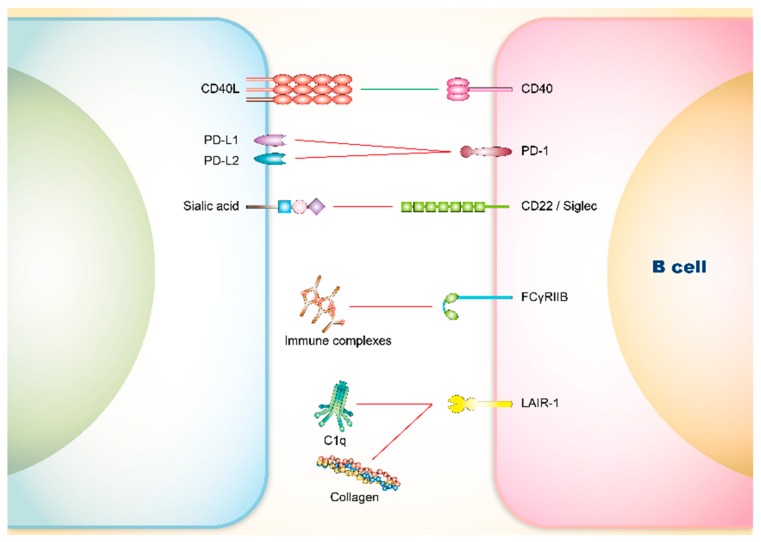
Co-signaling axes of B cells that attribute to SLE symptoms. B cell activity is also regulated by co-signaling axes. Co-stimulatory axes (green line) promote B cell activation, while co-inhibitory axes (red line) prevent B cell from activation. PD-1: programmed cell death protein 1; FcγRIIB: Fc fragment of IgG receptor IIb; LAIR-1: leukocyte-associated Ig-like receptor 1.

The presence of serum autoantibodies has been associated with SLE, supporting the view that a breakdown of self-tolerance in B cells and the production of antibodies against nuclear self-antigens play a central role in this disease [142]. Several co-signaling axes of B cells have been pointed out as being closely related to SLE (Figure 2).

### 3.1. Involvement of Co-Stimulatory Receptors on B Cells in SLE

#### CD40

Besides having been observed in T cells, two-signal activation has also been found in B cells, with the CD40-CD40L(CD154) axis, the interaction between the B cell-expressed CD40 and its binding partner CD40L, serving as the second signal required [143,144]. For rheumatic diseases that generate pathogenic autoantibodies, such as lupus, CD40L expressed on Tfh cells in germinal centers plays a key role in stimulating plasma cells with autoimmune specificities [145,146]. This may explain the effect of reductions in autoantibodies after treatment with anti-CD20-depletion drugs such as rituximab and ofatumumab [147,148,149]. Even though these short-lived antibody-producing plasmablasts and plasma cells are not directly targeted by anti-CD20 therapy, they are continuously derived from the CD20+ B cells via the induction of CD40L. Moreover, CD40L has been found to be ectopically expressed on B cells in lupus patients and lupus-prone mice [150,151]. In one study, CD40L-transgenic mice produce greater amounts of autoantibodies such as antinuclear Abs, anti-DNA Abs, and antihistone Abs with increasing age [151]. Moreover, almost half of the mice developed lupus-like disease characterized by glomerulonephritis with immune-complex deposition. Additionally, it was confirmed that anti-CD40L therapy in NZB/NZW.F1 mice decreases IgG anti-dsDNA Abs levels and delays the disease onset, likely by blocking both T cells from activation and B cells from class switching as well as somatic mutation [152]. In vivo study also revealed that anti-CD40L monoclonal Ab could block the over-expression of CD86 on B cells, which was found in patients with lupus [153], and further abrogate their anti-DNA Abs production [154]. However, clinical trials of the use of anti-CD40L to treat SLE have yet to yield positive results so far. 

There have been several clinical trials targeting this axis, including one in which an antibody targeting CD40L called IDEC-131 was used, with that trial failing to meet its desired endpoint [155]. Another trial of an antibody named BG9588 was terminated due to adverse effects such as hematuria and thromboembolic events [156]. Recently, a phase II clinical trial attempting to target CD40 by using a fully human IgG1 anti-CD40 monoclonal Ab, iscalimab (CFZ533), was started, and that trial is still undergoing [157]. It is noteworthy that the combinatorial treatment of anti-CD40L and anti-CTLA-4 for NZB/NZW.F1 mice has demonstrated synergistic effectiveness in delaying the onset of SLE by suppressing both autoreactive B and T cells [158,159], suggesting that combinatorial approaches have the potential to further enhance efficacy. Whether such synergistic effects could also be replicated in human SLE thus warrants further clinical studies.

### 3.2. Involvement of Co-Inhibitory Receptors on B Cells in SLE

#### 3.2.1. PD-1

The role of the PD-1-PD-L1/PD-L2 pathway in the development of lupus has largely been discussed with respect to T cells. However, the immune responses of B cells can also be regulated via this pathway [160,161]. In one study of SLE patients, the expression of CD19+PD-L1+B cells was enriched in the peripheral blood and correlated with disease-related laboratory parameters, clinical indicators (such as the Systemic Lupus Erythematosus Disease Activity Index), and Tfh cell populations [162]. CD19+PD-L1+B cells played an important role in activating the pathogenic T cell and B cell responses in SLE [162]. In another previous study, it was found that the activation of B cells via bacteria-derived oligodeoxynucleotides (CpG) alone or in combination with CD40/CD40L co-stimulation could significantly increase the expression of both PD-1 and PD-L1 on those B regulatory (Breg) cells [163]. Via PD-L1, the Breg cells could then limit the expansion and the function of PD-1+ Tfh cells, and down-regulate humoral immune responses [163]. Interestingly, these Breg cells are resistant to an anti-CD20-depletion drug, which thus generates a residual B-cell population that expresses high levels of PD-L1 and has potent T-cell-suppressive activity [161]. 

Recent research has further revealed that lupus B cells with enhanced PD-1 expression exhibit functionally reduced proliferation along with reduced PD-L1 up-regulation capacity upon stimulation by interleukin-2(IL-2)/IL-10, anti-B cell receptor (anti-BCR), CpG, and CD40L [164]. Moreover, PD-1 and PD-L1 interactions between Tfh and B cells help to maintain the stringency of affinity selection in germinal centers [165]. PD-L1-deficient B cells in a Sap^−/−^ mouse model developed an outgrowth of low-affinity or irrelevant antibodies following immunization, which contributed to the initiation and perpetuation of autoimmune disease [166]. 

#### 3.2.2. CD22 and Siglec

CD22/Siglec-2 and Siglec-G are membrane receptors that are restricted on B cells, and both belong to the sialic acid-binding Ig-like lectin (Siglec) family. CD22 presents throughout most of B2-cell development. It is firstly found on immature B cells, being most highly expressed on naïve B cells, but is then lost on plasmablasts and plasma cells [167]. It functions primarily as a negative regulator of BCR signaling, in addition to regulating Toll-like receptor signaling and the survival of B cells [168,169]. Siglec-G, on the other hand, is an important inhibitor on B1 cell subsets. These two B-cell Siglecs have been proven to have an important function in preventing autoimmunity, as double-deficient mice spontaneously develop a lupus-like phenotype with age that is characterized by antinuclear Ab development, lupus nephritis, and early death [170,171]. 

Epratuzumab, a humanized monoclonal Ab targeting CD22, can induce the rapid movement of CD22 into lipid draft without BCR activation, as well as the removal of BCR along with CD22 [172,173]. Therefore, it was thought to potentially be beneficial for the treatment of lupus. However, two large phase III studies yielded disappointing results in terms of the treatment responses of moderate and severe lupus patients [174,175]. Further analysis did show, however, a potential treatment response to epratuzumab among lupus patients with positive anti-Ro/La [176]. 

#### 3.2.3. FCγRIIB

FcγRIIB, one of the receptors for the Fc portion of IgG molecules (FcγRs), is the only inhibitory IgG Fc receptor that suppresses the activation of immune cells [176]. Unlike other FcγRs receptors, FcγRIIB possesses an immune receptor tyrosine-based inhibition motif (ITIM) in its cytoplasmic tail [177]. The tyrosine within the ITIM could be phosphorylated by the Src-family kinase Lyn when activated by antigen-antibody immune complex, and then transduce an inhibitory signal downwards [178]. Studies have revealed that this down-regulation is built on both increasing the BCR activation threshold and suppressing antigen internalization and presentation to T cells [179]. In previous studies, animal models consisting of FcγRIIb-deficient mice developed splenomegaly due to uninhibited expansion of B cells and formed lupus-like disease [177,180]. In humans, FcγRIIb-I232T, a polymorphic variant in which isoleucine at position 232 of FcγRIIb is replaced by threonine, is reported to be a risk allele for developing systemic lupus erythematosus. FcγRIIb-I232T shows a strong disease susceptibility in Southeast Asians, especially in the subgroups of lupus nephritis and male gender [181,182]. In addition, two ex vivo studies of PBMCs from SLE patients further identified reduced expression of FCγRIIB on memory B cells from SLE patients [183,184].

#### 3.2.4. Leukocyte Associated Immunoglobulin-Like Receptor (LAIR)-1

The leukocyte associated immunoglobulin-like receptor (LAIR)-1 is a transmembrane molecule belonging to the Ig superfamily which binds to different types of collagen [185]. Similar to FcγRIIB, it processes ITIM in the cytoplasmic domain, and thus causes the down-regulation of NK and T cell activation [186,187]. Within B cells, LAIR-1 cross-linking leads to down-regulation of the production of both immunoglobulins and cytokines in B cells [188]. Defective expressions of LAIR-1 on both B cells and plasmacitoid dendritic cells have previously been found in lupus patients, and such defective expressions can result in a lower inhibiting signal in Ig production after LAIR-1 and collagen interaction [189]. 

## 4. Co-Signaling Axes in Dendritic Cells (DCs) Relating to SLE 

DCs are critical sentinel cells that effectively link the innate and adaptive immune systems. Within lymph nodes and lymphoid organs, DCs present antigens to T cells, contributing to the induction of immunological tolerance and the expansion of protective pro-inflammatory immune responses [190]. In the last decade, studies of human and mice models have found that the aberrant activation of classical DCs (cDCs) or plasmacytoid DCs (pDCs), altered DC localization, and the functional impairment of DCs may contribute to the pathogenesis of SLE [4,5]. Intriguingly, reduced numbers of circulating pDCs and cDCs within the blood of patients with SLE have been reported, with those reduced numbers being correlated, in turn, with increased recruitment in the target tissues, possibly contributing to SLE progression [190,191,192,193].

As a distinct subset of DCs, pDCs can sense bacterial and viral pathogens, producing massive amounts of type I interferon (IFN) in response to infections [194]. 

Despite the nature of the strictly regulated type I IFN system, abnormal activation of it has been noted in a considerable proportion of patients with SLE, which was also found to be closely related to genetic variants that modulate this system [195].

**Figure 3:**
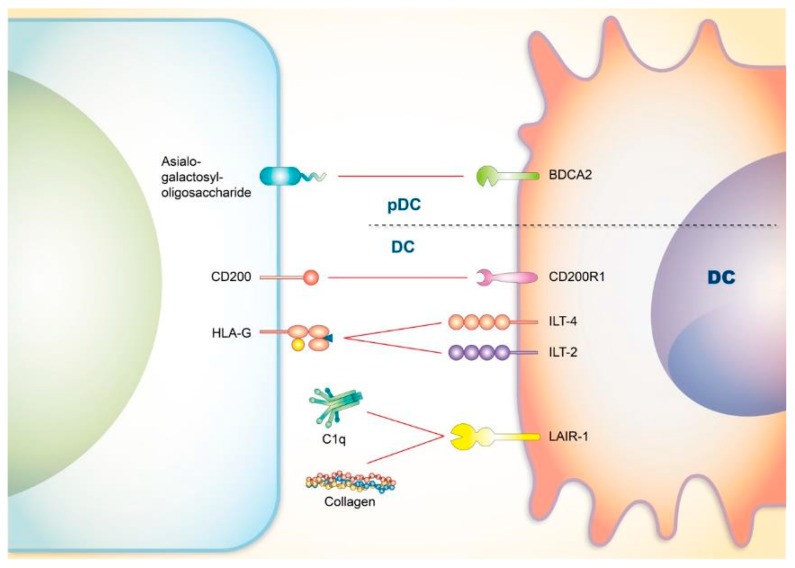
Co-signaling axes of DC involved in SLE. As the main producer of type I interferon, pDC is known to be regulated by co-inhibitory axes including BDCA2. Other co-inhibitory axes (red line) of DC also inhibit the immune responses that may be linked to SLE. DC: dendritic cell; pDC: plasmacytoid dendritic cell; BDCA2: Blood-derived dendritic cell antigen 2; ILT4: immunoglobulin-like transcripts 4; ILT2: immunoglobulin-like transcripts 2; LAIR-1: leukocyte-associated Ig-like receptor 1.

Type I IFN released by pDCs has been proposed to increase autoantibody formation by facilitating plasma cell differentiation [196,197]; in addition, heightened levels of type I IFN are observed in SLE patients, suggesting a positive role of pDCs in SLE development [198,199,200]. Since the therapeutic effects of some conventional medications against SLE, including hydroxychloroquine and glucocorticoids, may at least be partially contributed by their ability to downregulate the type I IFN system [201,202,203], targeting the co-signaling axis of pDCs could undoubtedly serve as a potential therapeutic strategy against SLE (Figure 3). 

### 4.1. CD200

With respect to co-signaling pathways affecting DCs, Li et al. reported that the aberrant functional status of CD200-CD200R1 signaling may contribute to the immunologic abnormalities of DC activity in SLE [108]. CD200+ apoptotic cells expressed by DCs and serum levels of soluble CD200 in SLE patients were significantly higher than those in healthy controls, whereas the expression of CD200R1 by CD4+ T cells and DCs was decreased. Heightened lymphocyte apoptosis and impaired phagocytic processing of apoptotic cells have been described as having impacts on the mechanisms of SLE [204,205,206]. Moreover, the early expression of apoptotic cells with increased CD200 expression was conjugated with their diminished binding and phagocytosis by DCs in SLE. In addition, this down-regulation of CD200R1 expression on DCs was also noted in lupus-prone mice along with elevated levels of anti-dsDNA Abs, which could be reversed by CD200-Fc treatment possibly through reducing the productions of IL-6 and IL-10 from DCs [107]. Therefore, augmenting the co-inhibitory effect of CD200R1 on DCs serves as a plausible strategy to be further explored.

### 4.2. Blood-Derived Dendritic Cell Antigen 2 (BDCA2) 

Blood-derived dendritic cell antigen 2 (BDCA2) is a pDC-specific receptor that inhibits the production of type-I IFN and other inflammatory cytokines when ligated [207]; also, BDCA2 signaling by pDCs restrains antigen processing and presentation to T cells [208]. In a previous study, BDCA2 was shown to recognize asialo-galactosyl-oligosaccharides with terminal galactose, which facilitates the binding of certain CD14+ monocyte-derived DCs and several human tumor cell lines [209]. As an inhibitory receptor of pDCs, the engagement of BDCA2 represents a feasible therapeutic target for inhibiting pDC-derived type-I IFN for the treatment of SLE.

Recently, Furie et al. reported on a phase 1b study demonstrating an approach to targeting the BDCA2 receptors of pDCs in SLE patients using a humanized monoclonal Ab (BIIB059); such targeting showed decreased type-I IFN expression and reduced immune infiltrates in cutaneous lesions with a favorable safety profile [210]. However, whether BIIB059 targeting of the type-1 IFN pathway will be effective in managing SLE in other organs remains unclear, which highlights the developing question of whether organ-specific approaches to lupus will be explored in future clinical trials [211].

### 4.3. Immunoglobulin-Like Transcript 4 (ILT4) and ILT2

On the other hand, immunoglobulin-like transcript 4 (ILT4) expressed by DCs is a well-characterized co-inhibitory receptor and recognizes human leukocyte antigen-G (HLA-G) [212]. Human leukocyte antigen-G (HLA-G) is a class I non-classical HLA molecule that plays an important regulatory role in the immune system during viral infections and some autoimmune diseases [213]. Paola Del Carmen Guerra-De-Blas et al. reported that significantly lower levels of ILT4-positive circulating pDCs and cDCs were detected in SLE patients; this diminished expression of ILT4 may contribute to a higher immunogenic phenotype of DCs in SLE [214]. It has also been reported that monocytes from psoriatic arthritis patients reveal downregulated expression of ILT4, demonstrating that alterations of these inhibitory receptors may not be exclusive to SLE patients but, rather, a common characteristic that SLE shares with other autoimmune conditions [215]. ILT2, on the other hand, also recognizes a broad range of classic class I MHC molecules including HLA–G. ILT2 was found to be downregulated in both pDCs and cDCs from SLE patients [216]. Moreover, as the common ligand of these axes, HLA-G was found to be diminished in monocytes from SLE patients, and a compromised ability of these monocytes to inhibit the proliferation of autologous lymphocytes was also revealed [217].

### 4.4. LAIR-1

To describe another co-signaling pathway affecting DCs in detail, C1q collagen-like region (CLR) engaging the leukocyte-associated Ig-like receptor 1 (LAIR-1; CD305), an inhibitory immunoreceptor expressed on pDCs, has been associated with the inhibition of Toll-like receptor activity and a suppressive effect on type-I IFN production from pDCs [218]. Classical pathway-mediated hypocomplementemia is a frequent feature in SLE patients and is often associated with the occurrence of autoantibodies (Abs) against C1q [219]; the high prevalence of anti-C1q Abs was previously strongly correlated with active lupus nephritis [220]. These findings indicate that complement C1q has a role in the pathogenesis of SLE. Notably, several studies have shown that LAIR-1 function and expression were impaired on B cells, monocytes, and DCs in SLE patients [189,221,222]. 

## 5. Co-Signaling Axes in Neutrophils Relating to SLE

Neutrophils, one of the important leukocytes primed towards the eradication of pathogens and the activation of inflammatory responses, have been linked to the pathophysiology of SLE. Neutrophils from SLE patients may display increased apoptosis, impaired ability to be removed by the C1q/calreticulin/CD91-mediated apoptotic pathway, defective phagocytosis, and altered oxidative metabolism [223,224,225]. 

Notably, the release of neutrophil extracellular traps (NETs) during a distinct process of cell death, known as NETosis, plays a crucial role in the tissue damage seen in patients with SLE [6]. First mentioned in 2004, NETs constitute an extracellular meshwork of DNA scaffolds bound to granular peptides that can entrap and kill microorganisms, and activate other immune cells [226,227]. 

In SLE patients, impaired degradation of NETs may result from the presence of DNase-1 inhibitors and anti-NET Abs [228,229]; Davis et al. conducted a phase Ib trial to investigate the safety and pharmacokinetics of recombinant human DNase-1 in patients with lupus nephritis, which showed well-tolerated results without significant adverse events [230]. Additionally, Steevels et al. observed that the ligation of signal inhibitory receptor on leukocytes-1 (SIRL-1), an inhibitory receptor expressed by neutrophils and monocytes, suppresses the release of NETs in SLE [231], and this inhibitory phenomenon was also found in both spontaneous and antibody-induced NETosis from neutrophils from SLE patients [232]. Moreover, this inhibitory effect of SIRL-1 is specific for NET formation, without having a dampening effect on the phagocytosis and ROS production that participate in intracellular microbial killing [233]. 

Besides SIRL-1, there are also other co-inhibitory axes that regulate NETosis, including siglec9, siglec5, and semaphorin4D. On the other hand, PILRα, which regulates the trafficking of neutrophils during inflammation, has drawn great attention recently, since targeting this axis potentially attenuates neutrophil influx and subsequent collateral tissue damage [234]. In mouse arthritis models, it was found that anti-PILRα mAb reduces inflammation and decreases the production of proinflammatory cytokines [235]. However, whether these novel findings can be utilized in therapies against SLE warrants further investigations. 

## 6. Conclusions 

To date, clinical trials targeting the co-signaling axes involved in SLE have yet to find great success, but further studies of these co-signaling axes are nonetheless warranted. Although the relationships between individual co-signaling pathways and SLE have been identified, their interactions with each other have yet to be clarified. In addition, combinatorial approaches may be reasonable approaches for overcoming autoimmunity, but current knowledge regarding the compensatory mechanisms between different axes may be insufficient to indicate the most potent combinations. Moreover, whether the observed changes in the expression of certain co-signaling molecules in patients with SLE are responses to or the pathogenic upstream of this complex disease requires further clarification. In line with these rationales, future studies targeting one or more co-signaling axes are encouraged to evaluate the responses with a more comprehensive approach given the essential complexity of immune responses.

## Figures and Tables

**Table 1:** Co-stimulatory axes involved in SLE.

Molecule	Expression	Ligand/Receptor	Possible Targeted Cells in SLE
CD80 and CD86	APCs	CD28	T cells
B7h	APCs	ICOS	T cells
OX40L	APCs	OX40	T cells
SLAMF6	T cells, B cells, and NK cells	SLAMF6	T cells
CD137L	APCs	CD137	T cells
CD40L	T cells	CD40	B cells

SLE: systemic lupus erythematosus; APCs: antigen presenting cells; ICOS: Inducible co-stimulator; SLAMF6: signaling lymphocyte activation molecule family 6; NK cells: natural killer cells.

**Table 2:** Co-inhibitory axes involved in SLE.

Molecule	Expression	Ligand/Receptor	Possible Targeted Cells in SLE
CD80 and CD86	APCs	CTLA4	T cells
PD-L1 and PD-L2	APCs	PD-1	T cells and B cells
VSIG-3	Unknown	VISTA	T cells
VISTA	APCs and T cells	VISTA receptor	T cells
CD200	B cells, eosinophils, pDCs and a subset of T cells	CD200R1	T cells, DCs, and neutrophils
CD155	DCs or macrophages	TIGIT	T cells and NK cells
Galectin-9	Cytoplasmic expression in most cell types.	TIM-3	T cells and macrophages
B7S1	APCs	B7S1 receptor	T cells
BTNL2	T cells, B cells, and macrophages	BTNL2 receptor	T cells
Unknown	APCs	B7S3	T cells
Sialic acid		Siglec-2/CD22	B cells
Immune complexes		FCγRIIB	B cells
Collagen (C1qCLR)		LAIR-1	B cells, DCs, and macrophages
Asialo-galactosyl-oligosaccharide		BDCA2	pDCs
HLA-G	Monocytes and trophoblasts	ILT-4	Myeloid cells, including monocytes, macrophages, dendritic cells, and granulocytes.
HLA-G	Monocytes and trophoblasts	ILT-2	T cells, B cells, DCs, and NK cells
VSTM1-L		SIRL-1	Neutrophils
Sialylated surface protein		PILR-α	Neutrophils

SLE: systemic lupus erythematosus; APCs: antigen presenting cells; CTLA-4: cytotoxic T-lymphocyte-associated antigen 4; PD-1: programmed cell death protein 1; VISTA: V-domain Ig suppressor of T cell activation; pDCs: plasmacytoid dendritic cells; DCs: dendritic cells; NK cells: natural killer cells; TIGIT: T-cell immunoreceptor with Ig and ITIM domains; TIM-3: T-cell immunoglobulin and mucin-domain containing-3; FcγRIIB: Fc fragment of IgG receptor IIb; LAIR-1: leukocyte-associated Ig-like receptor 1; BDCA2: Blood-derived dendritic cell antigen 2; ILT4: immunoglobulin-like transcripts 4; ILT2: immunoglobulin-like transcripts 2; SIRL-1: signal inhibitory receptor on leukocytes-1; PILR-α: paired immunoglobulin-like type 2 receptor.

**Table 3:** Current progress in co-signaling pathways targeted in clinical trials against SLE.

Medication	Target	Phase/Outcome	Clinical Trials.gov ID
Abatacept	CD80 and CD86	Phase III—terminated	NCT00430677
Abatacept	CD80 and CD86	Phase II—failed to meet endpoint	NCT00119678
Abatacept	CD80 and CD86	Phase II—failed to meet endpoint	NCT00774852
Abatacept	CD80 and CD86	Phase II—recruiting	NCT02270957
Abatacept	CD80 and CD86	Phase II—recruiting	NCT02429934
BMS-931699	CD28	Phase II—failed to meet endpoint	NCT02265744
AMG557	ICOSL	Phase I—acceptable safety profile	NCT00774943
JNJ-61610588	VISTA	Phase I—terminated	NCT02671955
CFZ533	CD40	Phase II—recruiting	NCT03656562
BG9588	CD40L	Phase II—terminated	Boumpas DT, et al. Arthritis Rheum. 2003.
IDEC-131	CD40L	Phase II—failed to meet endpoint	Kalunian KC, et al. Arthritis Rheum. 2002.
Dapirolizumab Pegol	CD40L	Phase II—unpublished	NCT02804763
Anti-CD40L	CD40L	Phase II—terminated	NCT00001789
Epratuzumab	CD22	Phase III—unpublished	NCT01408576
Epratuzumab	CD22	Phase III—terminated	NCT00111306
Epratuzumab	CD22	Phase III—terminated	NCT00383214
Epratuzumab	CD22	Phase III—withdrawn	NCT00382837
Epratuzumab	CD22	Phase III—failed to meet endpoint	NCT01262365
Epratuzumab	CD22	Phase III—failed to meet endpoint	NCT01261793
Epratuzumab	CD22	Phase II—unpublished	NCT01534403
Epratuzumab	CD22	Phase II—encouraging	NCT00624351
Epratuzumab	CD22	Phase II—encouraging	NCT00660881
Epratuzumab	CD22	Phase II—encouraging	NCT00383513
Epratuzumab	CD22	Phase II—terminated	NCT00113971
Epratuzumab	CD22	Phase I/II—acceptable safety profile	NCT01449071
Epratuzumab	CD22	Phase I—unpublished	NCT00011908
BIIB059	BDCA2	Phase II—active, not recruiting	NCT02847598
BIIB059	BDCA2	Phase I—acceptable safety profile	NCT02106897

SLE: systemic lupus erythematosus; ICOSL: Inducible co-stimulator ligand; VISTA: V-domain Ig suppressor of T cell activation; BDCA2: Blood-derived dendritic cell antigen 2.
